# Isolation of Two Novel Marine Ethylene-Assimilating Bacteria, *Haliea* Species ETY-M and ETY-NAG, Containing Particulate Methane Monooxygenase-like Genes

**DOI:** 10.1264/jsme2.ME11256

**Published:** 2012-02-04

**Authors:** Toshihiro Suzuki, Takamichi Nakamura, Hiroyuki Fuse

**Affiliations:** 1 Graduate School of Regional Environment Systems, Shibaura Institute of Technology, 307 Fukasaku, Minuma-ku, Saitama, Saitama 337–8570, Japan; 2 Chugai Technos Corp., 9–12 Yokogawa Shinmachi, Nishi-ku, Hiroshima, Hiroshima 733–0013, Japan; 3 College of Systems Engineering and Science, Shibaura Institute of Technology, 307 Fukasaku, Minuma-ku, Saitama, Saitama 337–8570, Japan

**Keywords:** short-chain alkene, *Haliea*, particulate methane monooxygenase (*pmo*)

## Abstract

Two novel ethylene-assimilating bacteria, strains ETY-M and ETY-NAG, were isolated from seawater around Japan. The characteristics of both strains were investigated, and phylogenetic analyses of their 16S rRNA gene sequences showed that they belonged to the genus *Haliea*. In C1–4 gaseous hydrocarbons, both strains grew only on ethylene, but degraded ethane, propylene, and propane in addition to ethylene. Methane, *n*-butane, and *i*-butane were not utilized or degraded by either strain. Soluble methane monooxygenase-type genes, which are ubiquitous in alkene-assimilating bacteria for initial oxidation of alkenes, were not detected in these strains, although genes similar to particulate methane monooxygenases (pMMO)/ammonia monooxygenases (AMO) were observed. The phylogenetic tree of the deduced amino acid sequences formed a new clade near the monooxygenases of ethane-assimilating bacteria similar to other clades of pMMOs in type I, type II, and Verrucomicrobia methanotrophs and AMOs in alpha and beta proteobacteria.

Ethylene occurs in the atmosphere at approximately 0.1–10 ppbv (41) and affects atmospheric chemistry and the global climate. It provides a sink for hydroxyl radicals and plays a key role in the production and destruction of ozone in the troposphere (10). Ethylene acts as a hormone in higher plants and is involved in the regulation of plant physiological processes. In addition, it can inhibit methane consumption activity and ammonium oxidation of soil (47). Higher plants generate ethylene from methionine via 1-aminocyclopropane-1-carboxylic acid (ACC). Many species of bacteria, yeasts, and molds produce ethylene from methionine via 2-keto-4-methylthiobutyric acid or from glutamic acid via 2-oxoglutarate (13).

Most isolates assimilating short-chain alkenes, ethylene, and/or propylene are terrestrial Gram-positive bacteria from the CMNR group or strains of *Xanthobacter*(37), such as *Rhodococcus rhodochrous* B-276 (formally, *Nocardia corallina*) (12, 39), *Xanthobacter autotrophicus* Py2 (17, 49), *Nocardioides* sp. JS614 (28), and *Mycobacterium*(15, 22). These strains oxidize alkenes by alkene monooxygenases to epoxyalkanes, which are further metabolized by epoxyalkane: coenzyme M transferase (5, 23). The alkene monooxygenases in these strains are soluble diion monooxygenases similar to soluble methane monooxygenases, phenol hydroxylases, toluene monooxygenases, and short-chain alkane mono-oxygenases (21, 25). The alkene monooxygenases are divided into two groups: those from *Xanthobacter autotrophicus* Py2 and the remainder. The former has an operon arrangement of an alpha subunit hydroxylase–ferredoxin–gamma subunit hydroxylase-coupling protein–beta subunit hydroxylase–reductase and the latter exhibit beta subunit hydroxylase-coupling protein–alpha subunit hydroxylase–reductase.

Ethylene is also produced in the sea, and ethylene concentrations in rock pools were recorded to range between 47.2 and 856.4 pmol L^−1^(3). A seasonal cycle was observed with a summer maximum and concentrations varied from 17 to 951 pmol L^−1^ in coastal waters. Ethylene concentrations in surface water vary in the range of 1.8–39.2 nL L^−1^ and generally show a vertical maximum at the pycnocline (approximately 100 m depth), where elevated concentrations of chlorophyll-*a*, dissolved oxygen, and nutrients were also found in the western Atlantic (36). Photochemical transformation of dissolved organic matter in surface water results in the production of ethylene (34). Some micro- and macroalgae, photosynthetic bacteria, and cyanobacteria produce ethylene, probably via ACC or acrylate from methionine (3, 27, 32). Few reports have examined the physiological effects of ethylene on algae, which is surprising given that ethylene may play a multifaceted role in algae, having driven the loss of chlorophyll-*a*(32) and having contributed to mastoparan-induced cell death in green algae (48). Nevertheless, no reports on ethylene-degrading marine micro-organisms currently exist.

We isolated ethylene-assimilating bacteria from seawater and investigated their characteristics, particularly those related to ethylene assimilation, to clarify the role of bacteria in ethylene circulation in the sea.

## Materials and Methods

### Growth conditions

A 5VM medium containing 100 mg NH_4_NO_3_, 10 mg KH_2_PO_4_, 2.5 mg Fe(III)EDTA, 2.75 mg vitamin B_12_, 2.5 mg biotin, 500 mg thiamine–HCl, 372 mg Na_2_EDTA, 0.25 mg CuSO_4_·5H_2_O, 5.75 mg ZnSO_4_·7H_2_O, 4.55 mg MnCl_2_·4H_2_O, 0.6 mg of CoCl_2_·6H_2_O, 0.27 mg (NH_4_)_6_Mo_7_O_24_·4H_2_O, and 5 mg yeast extract (Difco, Detroit, MI, USA) in 1 L filtered seawater (pH 8.1) was used to isolate and culture ethylene-assimilating bacteria. Ethylene was supplied by replacing 50% of the air in a culture vessel. Solid medium used for culturing 5VM media and 1% gellan gum. The culture was incubated at 25°C.

*Escherichia coli* strains were grown in Luria–Bertani (LB) broth containing 1% polypeptone, 0.5% yeast extract, and 1% NaCl medium supplemented with ampicillin (100 μg/mL) when necessary.

### Sampling and isolation of bacteria

Surface seawater was collected in 120-mL glass vials from several Japanese coasts during July 1998 and May 1999, and cultured with 5VM medium and ethylene. After growth was observed in the liquid medium, a portion of the broth was streaked onto solid medium and cultured with ethylene, and any visible colonies were transferred to liquid medium. This isolation procedure was repeated at least five times, at which point the purity of the isolated strain was confirmed by microscopic observation and by the lack of growth of other bacteria on Marine Agar 2216 (Difco) plates.

### Growth characteristics

The temperature range for growth was estimated by growing the isolates in liquid 5VM medium with ethylene at 4°C, 10°C, 20°C, 30°C, 37°C, and 45°C. To test the salt-dependence of growth, 0, 0.26, 2.6, 13, 26, 52, 79, or 132 g of NaCl was added to 1 L modified 5VM medium, in which seawater was replaced by 1.54 g CaCl_2_·2H_2_O, 100 mg KBr, 3 mg KF, 700 mg KCl, 30 mg H_3_BO_3_, 4.09 g K_2_SO_4_, 200 mg KHCO_3_, 17 mg SrCl_2_·6H_2_O, and 11.1 g MgCl_2_·6H_2_O in 1 L distilled water. The isolates were cultured in these media with ethylene.

To test their utilization of carbon sources with liquid 5VM medium, gaseous hydrocarbon was added by replacing 50% of the gas phase; alcohol was added at 1% and other carbon sources were added at 0.1%. We tested the nitrogen sources for the growth of isolates by adding them at 0.1% to liquid 5VM medium without NH_4_NO_3_ containing 0.1% sodium acetate as a carbon source. The growth of isolates was assessed by the turbidity of the media as well as protein concentrations, which were determined using a modified Lowry method (2).

### Oxidation of short-chain alkanes and alkenes by resting cells

The isolated ethylene-assimilating strains were cultured with 5VM medium and ethylene. The cultured cells were harvested by centrifugation at 7,000 × *g* and suspended in sterilized seawater at about one-tenth of the volume of their broth. The suspended cells were transferred to 13.5-mL glass vials, which were sealed with Teflon-lined rubber septa. Five or 10 μL nitrogen gas containing alkene and alkane gases was added to the vials. The vials were maintained at room temperature for 5 h, after which 0.1 mL of 5 N NaOH solution was added and the total hydrocarbon gases in the vials were extracted and analyzed using a gas chromatographic system (42) (GC-17A; Shimadzu, Tokyo, Japan) equipped with FID as a detector.

### Transmission electron microscopy (TEM)

Transmission electron microscopy analysis of the purified strains, which were cultured at 22°C for 20 days, was performed by negative staining using a JEM-2000EX (JEOL, Tokyo, Japan).

### Determination of DNA G+C content, cellular fatty acid profile, and ubiquinones

DNA G+C content, cellular fatty acid profile, and ubiquinones was determined by TechnoSuruga (Shizuoka, Japan).

### Isolation of total DNA and sequencing

Total DNA of ETY-M and ETY-NAG and DNA manipulations for *E. coli* was isolated according to Sambrook using the standard protocols (35). The 16S rRNA coding sequence was amplified by total DNA as a template and primers 9F (5′-GAGTTTGATCCTG GCTCAG-3′) (4) and 1510R (5′-GGTTACCTTGTTACGACTT-3′) (46). The *mmoX*-like gene was amplified by using mmoX206F (5′-ATCGCBAARGAATAYGCSCG-3′) (1) or mmoX1 (5′-CGGT CCGCTGTGGAAGGGCATGAAGCGCGT-3′) (29) as a forward primer, and mmoX886R (5′-ACCCANGGCTCGACYTTGAA-3′) (1) ormmoXr901(5′-TGGGTSAARACSTGGAACCGCTGGGT-3′)(38) as a reverse primer. The *pmoA*-like gene was obtained by PCR amplification using the forward primer A189f (5′-GGNGACTGG GACTTCTGG-3′) and reverse primer A682r (5′-GAASGCN GAGAAGAASGC-3′) (19). PCR amplification was performed using an S1000 Thermal Cycler (Bio-Rad, Hercules, CA, USA) using *Ex Taq* DNA polymerase (Takara Bio, Otsu, Japan) under the following conditions: for the 16S rRNA gene, a pre-denaturing step at 96°C for 2 min followed by 30 cycles at 96°C for 30 s, 55°C for 1 min, and 72°C for 2 min, and final elongation at 72°C for 7 min. PCR amplification of the *pmoA*-like gene and *mmoX*-like gene are described elsewhere (30). The amplified fragments of the 16S rRNA gene (1.5 kb) and the *pmoA*-like gene (0.5 kb) were cloned into pMD20, T-vector (Takara Bio), then transformed in *E. coli* DH5α as a host strain. DNA sequencing was carried out by cycle sequencing using the BigDye terminator v3.1 cycle sequencing kit (Applied Biosystems/Life Technologies, Carlsbad, CA, USA) with the ABI PRISM 310NT Genetic Analyzer (Applied Biosystems/Life Technologies). Accumulated sequencing data were analyzed with GENETYX-MAC software ver.15 (Genetyx, Tokyo, Japan).

### Phylogenetic analysis

Genetic analyses were conducted using 16S rRNA gene fragments of strains ETY-M and ETY-NAG, which were deposited in GenBank. Homology searches were conducted using the BLAST program (http://www.ncbi.nlm.nih.gov/BLAST/). The sequences were aligned using the CLUSTALW ver. 1.83 program. Phylogenetic trees were constructed using TreeViewX software with the neighbor-joining method. Bootstrap analysis with 100 trial replications was performed to determine the reliability of the clustering patterns.

### GenBank accession numbers

The GenBank accession numbers of the 16S rRNA gene sequences are ETY-M (AB646259) and ETY-NAG (AB646260).

## Results and Discussion

### Isolation, phenotypic characterization, and phylogenetic analyses of two ethylene-assimilating bacteria, strains ETY-M and ETY-NAG

Two strains of ethylene-assimilating bacteria, strain ETY-M and ETY-NAG, were isolated from seawater from Yakushima and Tokyo Bay, Japan, respectively. The two isolated strains exhibited attachment of growing cells to the inner wall of the culture vessel in the liquid medium but did not grow well on Marine Agar 2216 plates.

**Fig. 1:**
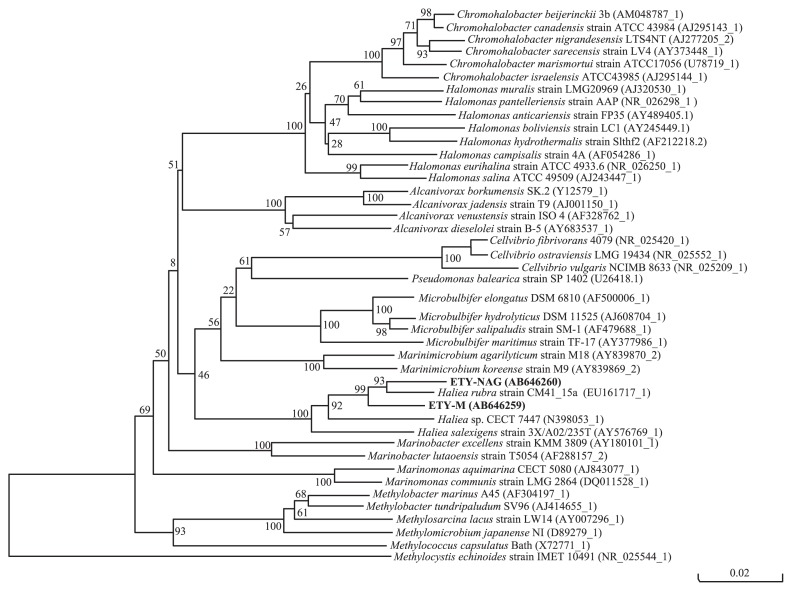
Phylogenetic tree of 16S rRNA genes constructed using a neighbor-joining dendrogram. *Methylocystis echinoides* served as an outgroup. Numbers to the right are accession numbers in the database. Scale bar indicates 0.02 substitutions per 100 base positions. Numbers at tree nodes are bootstrap values from 100 trials.

The 16S rRNA gene sequences were analyzed to examine the phylogeny of the strains. The analyses showed that the most closely related bacteria belonged to genus *Haliea* (Gammaproteobacteria). A BLAST search of strains ETY-M and ETY-NAG revealed similarities to the bacteria *Haliea* sp. MOLA 104 and *Haliea rubra* strain CM41_15a (43, 44), which were 97% and 96% identical, respectively. The phylogenetic tree showed that the two isolated strains were in the clade comprising *Haliea* spp. (Fig. 1).

Strains ETY-M and ETY-NAG were Gram-negative. Characteristics of both strains are shown in Table 1 with three type strains of *Haliea* spp. Strains ETY-M and ETY-NAG and *Haliea* spp. were isolated from marine samples and all strains needed NaCl for growth. All strains produced pigments although the colors were different.

The DNA G+C contents of strains ETY-M and ETY-NAG were 65.2 and 58.8 mol%, respectively, which is in accordance with the ranges reported for other *Haliea* species (Table 1). In addition, ETY-M and ETY-NAG exhibit ubiquinone Q-8, as do other *Haliea* species. The cellular fatty acid profile of strain ETY-M was determined, and five components of fatty acids were detected at concentrations greater than 1%: C18:1ω7c, 37.5%; (C16:1ω7c or C15:0 iso 2OH), 28.0%; C16:0, 21.0%; C14:0, 6.5%; C10:0 3OH, 3.2%. This profile is most similar to *H. rubra* in *Haliea* spp. Additional characteristics of both strains are compared with *Haliea* strains in Table 1.

With respect to carbon sources except for hydrocarbons, both strains showed good growth with the addition of acetate and pyruvate, as did the *H. mediterranea* strain 7SM29 (26), whereas strain ETY-NAG also assimilated maltose, sucrose, aspartate, and glutamate, and strain ETY-M also assimilated serine and alanine. As a nitrogen source, both strains utilized ammonium sulfate but not potassium nitrate. Furthermore, strain ETY-M utilized aspartate and arginine and strain ETY-NAG utilized arginine as nitrogen sources in addition to the amino acids they used for carbon sources. Therefore, on the basis of 16S rRNA gene sequence analyses as well as their physiological and biochemical characteristics, both strains were identified as *Haliea* spp.

### Strains ETY-M and ETY-NAG specifically assimilate ethylene

No strains of the genus *Haliea* have been reported to assimilate gaseous hydrocarbons, including ethylene. The assimilation of various gas hydrocarbons by strains ETY-M and ETY-NAG was investigated, and both strains were found to assimilate only ethylene, but not methane, ethane, propane, or propylene (Table 1). Only two strains, *R. rhodochrous* B-276 and *Mycobacterium* E20, have been reported to grow well on both alkanes and alkenes (37). *Mycobacterium* E20 utilized ethylene, but grew poorly on propylene and butene as well as ethane and higher alkanes (7), although ethylene was oxidized by the monooxygenases differently than for alkane oxidation (8). *R. rhodochrous* B-276 grew well on ethylene, propylene, propane, 1-butene, butane, and butadiene, but not on ethane or methane (14). *X. autotrophicus* Py2, as well as some other isolates, were able to use ethylene and propylene but not ethane, propane, or butane (45). Of the seven ethylene- or propylene-utilizing *Mycobacterium* strains tested, only one strain used both ethylene and propylene (9).

To test the conversion of gaseous hydrocarbons in both strains, the degradation of gaseous hydrocarbons by resting cells was performed with methane, ethane, propane, *i*-butane, *n*-butane, ethylene, and propylene (Table 2). Methane, *i*-butane, and *n*-butane were hardly degraded by both strains, while ethane, ethylene, and propylene were markedly degraded by both strains. Ethane, ethylene, and propylene were degraded to 0.48%, 0.02%, and 1.0%, respectively, by strain ETY-M, and to 0.06%, 0.15%, and 4.6%, respectively, by strain ETY-NAG. Propane was degraded a little more slowly than ethane, ethylene, and propylene by both strains, and was degraded to 9.3–58% by strain ETY-M and to 26% by strain ETY-NAG. Thus, C1 or C4 gases, such as methane, *i*-butane, and *n*-butane, were hardly degraded, while C2 or C3 gases, such as ethane, ethylene propane, and propylene, were degraded well by both strains. This indicated that ETY-M and ETY-NAG are able to specifically degrade C2 and C3 gas hydrocarbons, but not to assimilate them, except for ethylene.

Both strains grew on ethanol but not on 2-propanol. Strain ETY-M grew weakly on 1-propanol whereas strain ETY-NAG did not (Table 1). *X. autotrophicus* Py2 and *Mycobacterium* E20 were also able to assimilate ethanol. To clarify the ethylene degradation pathway in strains ETY-M and ETY-NAG, their metabolites and utilization should be investigated, along with other alkene-assimilating bacteria that epoxidize alkene for their growth.

Strains ETY-M and ETY-NAG were isolated from seawater using enrichment culturing with 50% ethylene. Both strains specifically assimilated ethylene in C1–4 gaseous hydrocarbons even though the ethylene concentration in seawater is very low. Ethylene appears to be produced by seaweed as well as some marine microorganisms. These ethylene-assimilating bacteria may coexist with ethylene producers such as members of the methylotrophic genus *Methylobacterium*, which are ubiquitous on plant surfaces and potentially dominate the phyllosphere population (6). The genus *Haliea* genus reportedly comprises several percent of the bacterial abundance in mangrove sediments and these species are sensitive to oil contamination (11). *Haliea* bacteria may interact with mangrove plants via ethylene, which is produced in abundance when plants are wounded; however, no information exists on the utilization of short-chain hydrocarbons by other *Haliea* spp.

### A particulate methane monooxygenase (*pmoA*)-like gene exists in ETY-M and ETY-NAG

Almost all alkene-assimilating bacteria, including ethylene-assimilating bacteria, carry soluble methane monooxygenase (sMMO)-like genes. To examine the existence of sMMO in both strains, we attempted to amplify the putative *mmoX* as a target gene, which encodes an α-subunit of the hydroxylase of the sMMO-like gene. The gene was amplified by all combinations of the four primers, i.e., mmoX206F or mmoX1 as a forward primer and mmoX886R or mmoXr901 as a reverse primer; however, no *mmoX*-like genes were detected in either strain (data not shown). These results suggest that the strains do not carry sMMO-like genes, but rather possess particulate methane monooxygenase (pMMO)-like genes because some pMMO-like enzymes related to short-chain alkane degradation were detected (16, 33). pMMO is a membrane-bound enzyme that requires copper for its activity. The pMMO gene cluster is composed of *pmoC*, *pmoA*, and *pmoB*, and pMMO is analogous to ammonia monooxygenase (AMO), whose gene cluster is composed of *amoC*, *amoA*, and *amoB*(19).

**Fig. 2:**
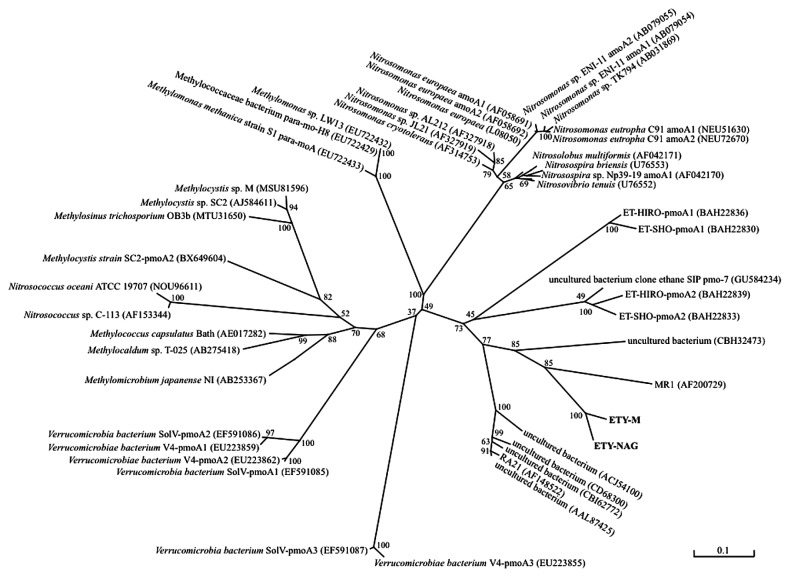
Phylogenetic tree of *pmoA*-like genes constructed using a neighbor-joining dendrogram. Numbers to the right are accession numbers in the database. Scale bar indicates 0.1 substitutions per amino acid position. Numbers at tree nodes are bootstrap values from 100 trials.

PCR amplification of the *pmoA*-like gene revealed the presence of the *pmoA*-like gene in both strains ETY-M and ETY-NAG. The BLAST search results for amplified *pmoA*-like gene sequences revealed similarities to *pmoA* (<54% similarity) and ammonia monooxygenase gene (*amoA*) (<63% similarity). Phylogenetic analysis of these genes indicated that *pmoA*-like genes of strain ETY-M and ETY-NAG were distant from other *pmo*-like genes, including methane-, ethane-, and ammonia monooxygenase; however, the genes of strains ETY-M and ETY-NAG clustered nearest to the genes of putative ethane oxidizers that were retrieved from marine sediment by the SIP technique (33) as well as the genes of ethane-assimilating strains ET-HIRO and ET-SHO in GenBank (Fig. 2). The *pmoA*-like genes of strains ETY-M and ETY-NAG were particularly analogous to those of *Nitrosospina* and *Nitrosomonas*, which are beta-proteobacteria (31), compared to those of methanotrophs. In the phylogenetic tree, the *pmoA*-like genes of both strains formed a new branch of *pmoA*-like genes of ethylene-assimilating bacteria. Some environmental *pmoA*/*amoA*-like clones, which were reported as the RA21 cluster/group (24, 40), were placed near this clade (Fig. 2). Clone RA21 was retrieved from a beech forest soil sample in Denmark and was described as not being placed in any known group of *pmo* or *amo* sequences (20). Clone MR1 was also retrieved from forest soil near Marburg, Germany, and was described as a putative ammonium oxidizer (18). These genes could be specific to bacteria that assimilate short-chain hydrocarbons, particularly ethylene or ethane.

This is the first report to demonstrate that some strains of *Haliea* have the ability to degrade gaseous hydrocarbons and that *pmoA*-like genes are found in isolated bacteria besides methanotrophs and ammonia oxidizers. The genes encoding pMMO or AMO clusters are in the order ‘CAB’ with the exception of archaean AMO and pXMO, which are putatively involved in ammonium oxidation in some methanotrophs, although their specific functions are not clear (40). The gene clusters of *pmo*-like genes in strains ETY-M and ETY-NAG and their relation to ethylene degradation and these genes will be clarified in future studies.

## Figures and Tables

**Table 1:** Characterization of ETY-M, ET-NAG, and other *Haliea* species

Characteristic	Strains				

	ETY-M	ETY-NAG	3X/A02/235	CM41_15a	7SM29T
Cell morphology	Short Rods	Short Rods	Straight Rods	Straight Rods	Short Rods
Cell dimensions (μm)	0.4–0.45 × 1.2–1.3	0.75–1.0 × 0.5–0.6	0.3–0.7 × 1.3–1.9	0.5 × 2.7	0.4–0.5 × 1.1–1.3
Colony color (agar medium)	Purple (5VM)	Yellow (5VM)	Cream (MA)	Red (MA)	Yellow (MA)
Growth of nutrient agar (MA)	−	−	+	+	+
Flagella	−	+	+	−	+
DNA G+C content (mol%)	65.2	58.8	61.4	64.8	62.1
Growth temperature range (°C)	20–37	20–30	10–37	15–44	15–40
Optimum	30	30	25–30	30	28
Salinity range (g L^−1^)	13.2–52.7	26.4–52.7	7–70	7–42	3.5–150
Optimum	13.2	26.4	40	35	unknown
Growth substrate					
Glucose	−	−	−	+	−
Maltose	−	+	−	(+)	−
Sucrose	−	+	−	−	−
Arabinose	−	−	−	−	−
Xylose	−	−	N.A.	N.A.	−
Fructose	−	−	−	(+)	−
Mannose	−	−	(+)	(+)	−
Cellobiose	−	−	−	−	(+)
Mannitol	−	−	−	N.A.	−
Citrate	−	−	−	+	−
Succinate	−	−	+	−	−
Gluconate	−	−	N.A.	N.A.	−
Pyruvate	+	+	+	−	+
Acetate	+	+	−	−	+
Glycerol	−	(+)	+	−	−
Alanine	+	−	−	−	+
Glutamate	−	+	(+)	−	+
Aspartate	−	+	+	−	+
Serine	+	−	−	−	−
Gas hydrocarbon utilization of					
Methane	−	−	N.A.	N.A.	N.A.
Ethane	−	−	N.A.	N.A.	N.A.
Propane	−	−	N.A.	N.A.	N.A.
Ethylene	+	+	N.A.	N.A.	N.A.
Propylene	−	−	N.A.	N.A.	N.A.
Methanol	−	−	N.A.	N.A.	N.A.
Ethanol	+	+	N.A.	N.A.	N.A.
1-Propanol	(+)	−	N.A.	N.A.	N.A.
2-propanol	−	−	N.A	N.A	N.A

MA, marine agar 2216; +, positive; −, negative; (+), weakly positive; N.A., data not available.

**Table 2:** Degradation of various alkanes and alkenes by ethylene-grown ETY-M and ETY-NAG

Substrates	Residual gas hydrocarbons (%)		

	ETY-M (A)	ETY-M (B)	ETY-NAG
Methane	121 (0.11)	ND	98 (0.01)
Ethane	0.38 (0.05)	0.48 (0.07)	0.06 (1 × 10^−4^)
Ethylene	ND	0.02 (0.03)	0.15 (1.3 × 10^−4^)
Propane	58 (0.1)	9.3 (0.03)	26 (0.02)
Propylene	ND	1.0 (0.008)	4.6 (0.04)
*i*-Butane	122 (0.06)	91 (0.05)	102 (0.01)
*n*-Butane	118 (0.09)	90 (0.05)	102 (0.02)

For ETY-M, experiments ETY-M (A) and ETY-M (B) were done simultaneously but separately; 5 μL of mixed gas (A) containing 1% each of methane, ethane, propane, *i*-butane, and *n*-butane was injected into a vial for ETY-M (A), and 5 μL of mixed gas (B) containing 1% each of ethane, ethylene, propane, propylene, *i*-butane, and *n*-butane was injected into a vial for ETY-M (B). For ETY-NAG, 5 μL of mixed gas (A) and (B) was injected into a vial. Total protein concentrations of ETY-M and ETY-NAG, were 0.52 mg L^−1^ and 0.07 mg L^−1^, respectively. ND, not determined. Values given in parentheses are standard deviations for three replicates in ETY-M (A) and ETY-M (B) and are the half differences of two replicates in ETY-NAG.
